# Ambient temperature regulates root circumnutation in rice through the ethylene pathway: transcriptome analysis reveals key genes involved

**DOI:** 10.3389/fpls.2024.1348295

**Published:** 2024-03-08

**Authors:** Zeping Cai, Yinuo Dai, Xia Jin, Hui Xu, Zhen Huang, Zhenyu Xie, Xudong Yu, Jiajia Luo

**Affiliations:** ^1^ School of Tropical Agriculture and Forestry, Hainan University, Hainan, China; ^2^ Tropical Crops Genetic Resources Institute, Chinese Academy of Tropical Agricultural Sciences, Haikou, Hainan, China

**Keywords:** ambient temperature, root tip, reorientation, transcriptome sequencing, gene screening, weighted gene co-expression network analysis

## Abstract

Plant roots are constantly prepared to adjust their growth trajectories to avoid unfavorable environments, and their ability to reorient is particularly crucial for survival. Under laboratory conditions, this continuous reorientation of the root tip is manifested as coiling or waving, which we refer to as root circumnutation. However, the effect of ambient temperature (AT) on root circumnutation remains unexplored. In this study, rice seedlings were employed to assess the impact of varying ATs on root circumnutation. The role of ethylene in mediating root circumnutation under elevated AT was examined using the ethylene biosynthesis inhibitor aminooxyacetic acid (AOA) and the ethylene perception antagonist silver thiosulfate (STS). Furthermore, transcriptome sequencing, weighted gene co-expression network analysis, and real-time quantitative PCR were utilized to analyze gene expressions in rice root tips under four distinct treatments: 25°C, 35°C, 35°C+STS, and 35°C+AOA. As a result, genes associated with ethylene synthesis and signaling (*OsACOs* and *OsERFs*), auxin synthesis and transport (*OsYUCCA6*, *OsABCB15*, and *OsNPFs*), cell elongation (*OsEXPAs*, *OsXTHs*, *OsEGL1*, and *OsEXORDIUMs*), as well as the inhibition of root curling (*OsRMC*) were identified. Notably, the expression levels of these genes increased with rising temperatures above 25°C. This study is the first to demonstrate that elevated AT can induce root circumnutation in rice via the ethylene pathway and proposes a potential molecular model through the identification of key genes. These findings offer valuable insights into the growth regulation mechanism of plant roots under elevated AT conditions.

## 1 Introduction

Plant roots encounter a variety of challenges in the soil. The root tips can perceive various stimuli, such as gravity, temperature, light, obstacles, and chemicals, to adjust their growth trajectories and avoid unfavorable environments (Roy and Bassham, 2014). Therefore, the ability of root reorientation is particularly important in nature. Under laboratory conditions, the sustained reorientation of root tips is manifested as coiling or waving, which we refer to as root circumnutation. Root circumnutation is regulated by endogenous hormones such as auxin, ethylene, brassinosteroids (BRs), and jasmonic acid (JA) (Woods et al., 1984; Okada and Shimura, 1990; Muller et al., 1998; Jiang et al., 2007; Cai et al., 2018). Additionally, environmental factors such as light, obstacles, gravity, and nutrient substances affect root circumnutation through endogenous hormone network (Okada and Shimura, 1990; Chen et al., 2015; Chai et al., 2020; Taylor et al., 2021).

The growth of plant roots is significantly influenced by ambient temperature (AT). For example, higher AT can promote primary root elongation and lateral root formation in *Arabidopsis thaliana* (Gray et al., 1998; Martins et al., 2017). The primary roots of maize (*Zea mays*) exhibit thermotropism, which is most evident at 15°C among lower temperatures (Fortin and Poff, 1990, 1991). Compared to 23°C, *Arabidopsis* roots exhibit enhanced positive gravitropism at 29°C (Hanzawa et al., 2013), while the gravitropic response of rice (*Oryza sativa*) root tips is significantly inhibited at 4°C (Du et al., 2013). Rice root tips exhibit weakened negative phototropism at 40°C, but the growth rate is significantly inhibited due to adverse effects on root metabolism (Wang et al., 2002). Moreover, at an inclined gel surface with temperature of 28°C, *Arabidopsis* roots form wave-like curvatures at a higher rate and with greater angles than at a temperature of 23°C (Thompson and Holbrook, 2004). These findings suggest that AT can affect the directional growth of roots, which prompts us to further explore its relationship with root circumnutation.

Ethylene, an important gaseous plant hormone, plays a critical role in regulating root circumnutation. Ethylene has been shown to induce root circumnutation in a concentration-dependent manner in plants, including species like tomato (*Lycopersicon esculentum*), *A. thaliana*, and rice (Woods et al., 1984; Buer et al., 2003; Cai et al., 2021b). Factors such as AT, light, obstacles, and BRs have all been demonstrated to promote ethylene biosynthesis and signaling (Eliasson and Bollmark, 1988; Okamoto et al., 2008; Fei et al., 2017; Lee and Yoon, 2018). Interestingly, apart from AT, all of these factors have also been shown to induce root circumnutation. Root circumnutation induced by light, obstacles, and BRs can be completely abolished by inhibitors of ethylene synthesis and/or action, indicating that their regulation of root circumnutation is dependent on the ethylene pathway (Chen et al., 2015; Cai et al., 2018; Taylor et al., 2021). However, it remains unclear whether AT can also regulate root circumnutation through the ethylene pathway.

On the gene level, studies have revealed that auxin-related genes play a significant role in root circumnutation, such as *YUCCA*, *ARABIDOPSIS THALIANA ATP-BINDING CASSETTE B* (*ABCB*), and *NITRITE TRANSPORTER 1*/*PEPTIDE TRANSPORTER FAMILY* (*NPF*). *YUCCA* encodes the rate-limiting enzyme in the process of auxin synthesis, while *AtABCB19*, *AtABCB4*, and *AtNPF7.3* encode auxin carriers. When vertically cultured, *Arabidopsis* mutants *yucca8*, *abcb19*, *abcb4 abcb19*, and *npf7.3* exhibit root circumnutation phenotypes (Lewis et al., 2007; Wu et al., 2015; Watanabe et al., 2020). In addition, it has been demonstrated that root circumnutation is a process driven by differential cell elongation on the upper and lower sides of the turning root (Su et al., 2017; Cai et al., 2018; Taylor et al., 2021). This indicates that genes involved in regulating cell elongation and cell wall loosening are implicated in this process. Moreover, *Oryza sativa Root Meander Curling* (*OsRMC*), which encodes a Cysteine-rich repeat receptor-like protein, was initially discovered in JA-induced root circumnutation and acts as a negative regulator of this process (Chen, 2001; Jiang et al., 2007). Specifically, the primary roots in *RNAiOsRMC* rice plants coiled as they touched the bottom of culture bottles in light (Jiang et al., 2007). Thus, the expression changes of the aforementioned genes in root circumnutation warrant further investigation.

In previous studies, we utilized rice as a material and established an effective system for studying root circumnutation (Cai et al., 2018; Cai et al., 2021a, b). Building upon this work, the present study continues to utilize rice seedlings as the material to investigate the impact of different ATs on root circumnutation. The role of ethylene in regulating root circumnutation under AT was examined by applying the ethylene biosynthesis inhibitor aminooxyacetic acid (AOA) and the ethylene perception antagonist silver thiosulfate (STS). Additionally, transcriptome sequencing, weighted gene co-expression network analysis (WGCNA), and real-time quantitative PCR (qRT-PCR) were employed to analyze gene expression in rice root tips under four treatments: 25°C, 35°C, 35°C+STS, and 35°C+AOA. Using these analytical methods, we identified responsive genes that are induced by AT via the ethylene pathway. Furthermore, the expression changes of these genes with increasing temperature were also investigated. The aforementioned findings provide valuable insights into the growth regulation mechanism of plant roots under elevated ATs.

## 2 Materials and methods

### 2.1 Plant materials and chemicals

The rice variety employed in this study was ‘Xiuzhan 15’ (Cai et al., 2021a). L-aminocyclopropane-1-carboxylic acid (ACC, CAS: 22059-21-8), Ethephon (ETP, CAS: 16672-87-0), and AOA (CAS: 645-88-5) were sourced from Aladdin, Ron, and Sigma companies, respectively. According to the solubility of the reagents in water at 4°C and the convenience of dilution, ACC was prepared as a 0.1 M mother solution, ETP as a 0.25 M mother solution, and AOA as a 1 mM mother solution, all using distilled water, and stored at 4°C (Cai et al., 2021b). A 0.92 mM STS solution was freshly prepared by mixing equal volumes of 1.28 mM Na_2_S_2_O_3_·5(H_2_O) and 1.84 mM AgNO_3_.

### 2.2 Experimental methods

#### 2.2.1 Sterilization and germination of seeds

Following the method of Cai et al. (2018), rice seeds were sterilized and germinated. Rice seedlings with uniformly elongated roots (1-2 mm in length) were selected for further experiments.

#### 2.2.2 Effect of ambient temperature on root growth

Four ATs were set: 20°C, 25°C, 30°C, and 35°C. In each culture dish (Ø 150mm), 30 ml of distilled water was added as the culture solution. Subsequently, at least 35 rice seedlings were planted and cultured for 1 day under a photosynthetic photon flux density of 73.6 μmol/s/m^2^. Root growth is known to be slow at 20°C; therefore, to exclude the possibility that short root length was the cause of any observed lack of root circumnutation, we extended the culture time to 5 days. Root circumnutation ratios and root lengths were measured using the method described by Cai et al. (2018). Each treatment was repeated at least five times. The least significant difference (LSD) test was employed for statistical analysis. Throughout the experiment, consistent lighting conditions and statistical methods were maintained.

#### 2.2.3 Effect of ethylene on root growth

The experiments were conducted at 25°C. According to Cai et al. (2021b), 100 µM ACC or 50 µM ETP was used as the rice culture solution (represented as 25°C+ACC and 25°C+ETP, respectively). After 2 days of cultivation, the rice root circumnutation ratios and root lengths were measured.

#### 2.2.4 Effect of ethylene inhibitor on the root circumnutation induced by higher AT

The experiments were conducted at 35°C. According to Cai et al. (2018), 100 µM AOA or 0.92 mM STS was used as the rice culture solution (represented as 35°C+AOA, and 35°C+STS, respectively). After 1 or 2 days of cultivation, the root circumnutation ratios and root lengths of rice were measured.

#### 2.2.5 Collection of root tip tissue samples

According to Cai et al. (2021a), rice root tips were collected after being cultured for 1 day under the following conditions: 25°C, 35°C, 35°C+STS, and 35°C+AOA (later referred to as the first group treatment); and 20°C, 25°C, 30°C, and 35°C (later referred to as the second group treatment). As the root tips curled around once with a length of approximately 0.5 cm, all collected root tips in this study measured 0.5 cm in length. Approximately 500 root tips (about 0.5 g) were collected for each sample and stored in liquid nitrogen. Each treatment had three biological replicates.

#### 2.2.6 RNA extraction, library construction, and transcriptome sequencing

Total RNA was extracted from each sample using the CTAB method (Sun et al., 2003). mRNAs were enriched using magnetic beads with oligo (dT) and fragmented for cDNA synthesis and library construction. Transcriptome sequencing was performed using BGISEQ for the first group treatment and the DEBSEQ platform for the second group treatment. The clean reads were saved in FASTQ format after removing low-quality reads (Cock et al., 2010). HISAT (Kim et al., 2015) was used to align the clean reads to the rice genome sequence (*O. sativa* Japonica Group, GCF_001433935.1_IRGSP-1.0), and RSEM (Li and Dewey, 2011) was used to calculate the expression levels of each gene in each sample in terms of fragments per kilobase million (FPKM). All the above procedures were conducted by the Beijing Genomics Institute (BGI). The sequencing data have been deposited in the SRA database under the accession numbers PRJNA788706 for the first group treatment and PRJNA1020455 for the second group treatment.

#### 2.2.7 Quality assessment of transcriptome sequencing

The clean reads of each sample were analyzed for sequencing saturation by using the Perl software. Utilizing the expression data of all genes, we performed cluster analysis, Pearson correlation analysis, and principal component analysis (PCA) on the samples. Specifically, the ‘pheatmap’ package in R was employed for cluster analysis, while the ‘cor’ and ‘prcomp’ functions within R software facilitated Pearson correlation analysis and PCA, respectively, as outlined in Cai et al. (2021b).

#### 2.2.8 Screening of differentially expressed genes

In this study, a total of 3 comparison groups were set up: 25°C vs 35°C, 35°C vs 35°C+STS, and 35°C vs 35°C+AOA. Differentially expressed genes (DEGs) were detected and analyzed by BGI, following the methodology outlined by Wang et al. (2010). Genes exhibiting an absolute log_2_ FoldChange of ≥1 and a *Q*-value of ≤0.001 were considered as differentially expressed and thus defined as DEGs.

#### 2.2.9 Weighted gene co-expression network analysis

The selected DEGs were analyzed using the ‘WGCNA’ package in R, following the methodology outlined by Zhang and Horvath (2005) and Langfelder and Horvath (2008). For this analysis, the parameters were set as follows: ‘sft$powerEstimate’ was set to 30, and ‘mergeCutHeight’ was set to 0.25. Subsequently, the module that showed the highest correlation with root circumnutation was chosen for further investigation.

#### 2.2.10 Gene ontology annotation and classification

The selected DEGs were annotated and classified utilizing the Blast2GO (v5.1) software (https://www.blast2go.com). This software aligned the sequences of DEGs using blastx (*E*-value ≤ 0.001) against the NCBI non-redundant protein database (Nr database), as described by Conesa et al. (2005) and Götz et al. (2011). Within the Gene Ontology (GO) system, hierarchical relationships among nodes were represented as a directed acyclic graph, wherein lower-level nodes denoted more specific functions. Notably, the color intensity of the nodes corresponded to the nodescore value; deeper hues signified higher nodescore values, indicating a stronger association with the respective GO term.

#### 2.2.11 Gene co-expression network visualization

Following the methodology outlined in Franz et al. (2016), we visualized the co-expression relationships among the selected DEGs using Cytoscape software (version 3.7.1). In our visualization, the size of each node corresponds to the degree value of the respective gene, indicating its connectivity within the network. Similarly, the thickness of the edges connecting the nodes represents the weight value between genes, reflecting the strength of their co-expression relationships.

#### 2.2.12 Real-time quantitative polymerase chain reaction

Real-time quantitative polymerase chain reaction (RT-qPCR) was performed to validate the expression of selected DEGs under different treatments. The primers were synthesized by BGI (**Supplementary Table 1**). The housekeeping genes *OsActins* (LOC4334702 for the first group treatment, LOC4331146 for the second group treatment) that expressed relatively stable in samples of the transcriptomes were chosen as reference genes. The 2^-ΔΔCt method (Pfaffl, 2001) was used for relative quantification. Three biological replicates were performed for each treatment, and each biological replicate was tested in triplicate.

## 3 Results

### 3.1 Higher ambient temperature induces root circumnutation in rice

**Figure 1:**
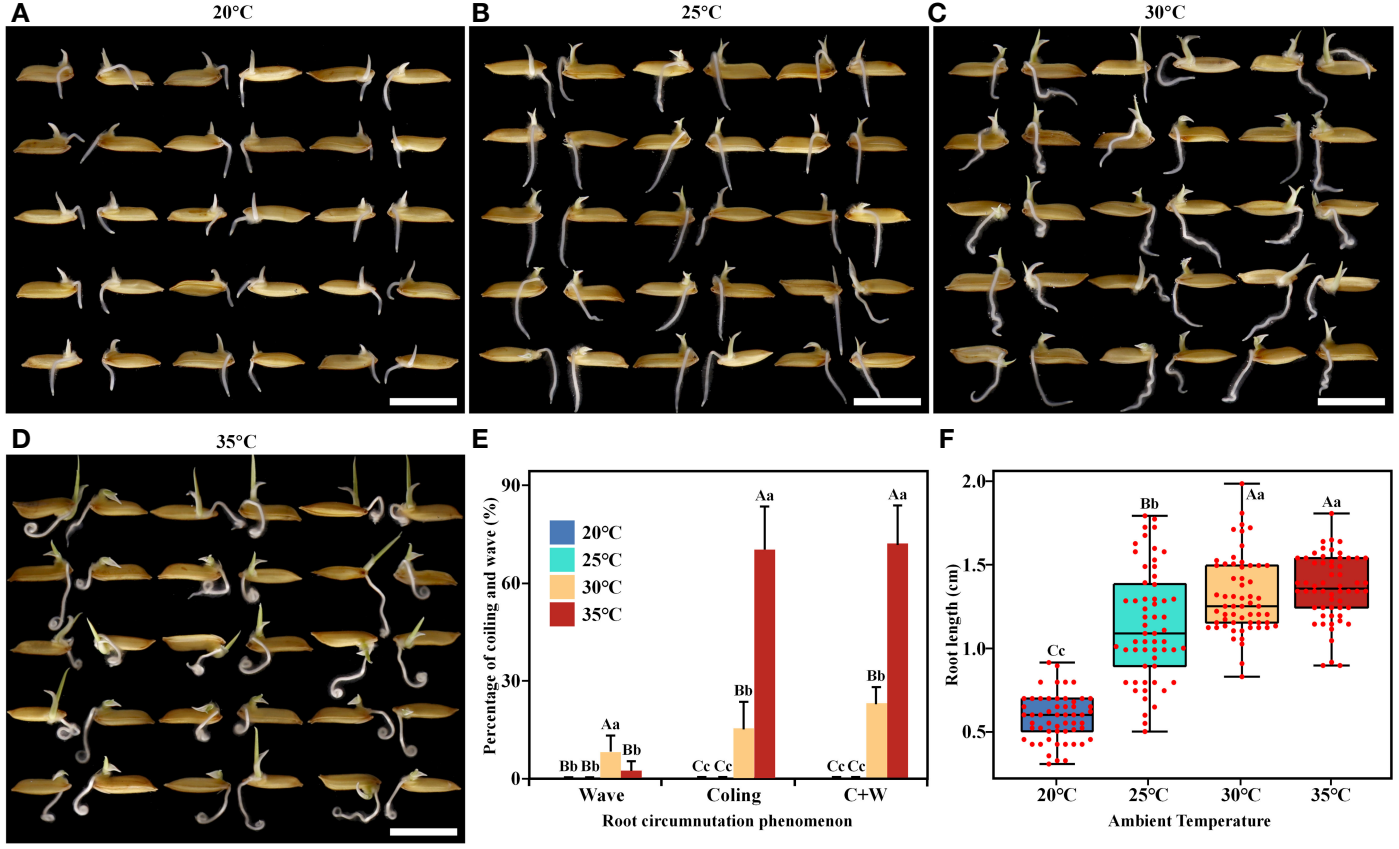
Effects of different ambient temperatures on rice root growth. **(A-D)** Rice seedlings grown at 20°C **(A)**, 25°C **(B)**, 30°C **(C)**, and 35°C **(D)**, respectively. Cultivation time was 1 day, scale bar = 1 cm. **(E, F)** Root circumnutation percentages **(E)** and root lengths **(F)** at the four different ambient temperatures tested. The total percentage of coiling and wave is represented by ‘C+W’. Different lowercase letters indicate significant differences (*p*<0.05), while different uppercase letters indicate highly significant differences (*p*<0.01) between corresponding treatments. This notation applies to all subsequent figures.

To investigate the regulatory role of AT on rice root circumnutation growth, four temperature gradients (20°C, 25°C, 30°C, and 35°C) were set up. When AT was below 25°C, the ratios of root circumnutation were extremely low (**Figures 1A, B**). When AT was above 30°C, root circumnutation occurred, forming wave and coiling (**Figures 1C, D**). At 30°C, the ratios of wave, coiling, and coiling+wave (hereafter referred to as ‘C+W’) were 7.7%, 15.0%, and 22.7%, respectively (**Figure 1E**), and the median root length was 1.25 cm (**Figure 1F**). At 35°C, the degree of root circumnutation reached its maximum, with ratios of wave, coiling, and C+W at 1.7%, 70.0%, and 71.7%, respectively (**Figure 1E**), and the median root length was 1.37 cm (**Figure 1F**).

Root growth was slow at 20°C. To rule out the possibility that circumnutation did not occur due to shorter root length, we extended the culture time. The results showed that the root length of 2.85 cm cultivated at 20°C for 5 days greatly exceeded that of 1.37 cm cultivated at 35°C for 1 day, but the ratios of root circumnutation were still 0 (**Supplementary Figure 1**). Root circumnutation can only be induced at higher AT.

### 3.2 The induction of root circumnutation by higher AT is dependent on ethylene pathway

Firstly, to investigate the role of ethylene in the induction of root circumnutation, 100 µM ACC or 50 µM ETP was applied alone at 25°C (hereafter referred to as 25°C+ACC and 25°C+ETP, respectively). Results showed that both 25°C+ACC and 25°C+ETP treatments induced rice root circumnutation, with wave ratios of 8.6% and 11.5%, coiling ratios of 24.1% and 20.1%, and C+W ratios of 32.7% and 31.6%, respectively (**Supplementary Figure 2**). These phenotypes were consistent with previous results from our research group (Cai et al., 2021b).

**Figure 2:**
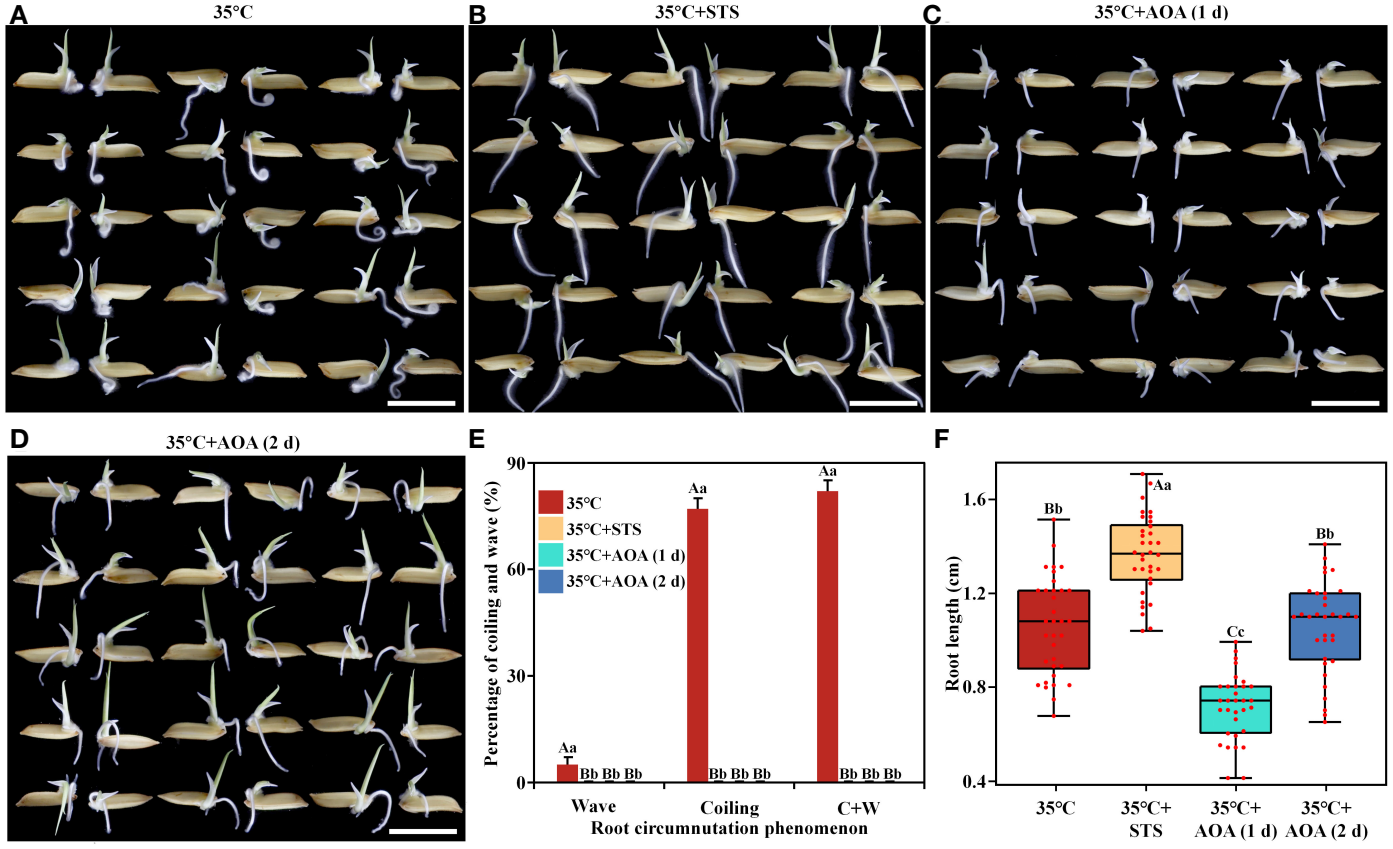
Ethylene inhibitors abolish the root circumnutation induced by higher ambient temperature. **(A-D)**: Root growth of rice under 35°C **(A)**, 35°C+STS **(B)**, and 35°C+AOA **(C, D)** treatments (1 d for **A**-**C** and 2 d for **D**); **(E, F)** Root circumnutation percentages **(E)** and root lengths **(F)** under these treatments. Scale bar = 1 cm.

Secondly, 0.92 mM STS or 100 µM AOA was applied at 35°C (hereafter referred to as 35°C+STS and 35°C+AOA, respectively) to investigate whether ethylene inhibitors could eliminate root circumnutation induced by higher AT (**Figures 2A-D**). The results showed that more than 80% of rice roots exhibited circumnutation, and the median root length was 1.08 cm after 1 day of culture at 35°C. However, the root circumnutation ratios were both 0, and the median root lengths were 1.38 cm and 0.75 cm, respectively, after 1 day of culture in 35°C+STS and 35°C+AOA treatments (**Figures 2E, F**). To exclude the possibility that roots did not exhibit circumnutation in the 35°C+AOA treatment after 1 day due to their shorter length, the culture time was extended to 2 days. At this time point, the median root length in the 35°C+AOA treatment reached 1.10 cm, but the root circumnutation ratios remained 0 (**Figures 2E, F**). These results indicate that the induction of root circumnutation by higher AT can be completely eliminated by ethylene synthesis or action inhibitors.

In conclusion, higher AT can induce rice root circumnutation, and this process is dependent on ethylene synthesis and signaling.

### 3.3 Quality assessment of transcriptome sequencing

Transcriptome sequencing was conducted on 12 samples, encompassing three biological replicates for each of the four treatments (25°C, 35°C, 35°C+STS, and 35°C+AOA). Per sample, an average of 6.78 Gb of data was generated. Post-filtering of raw reads yielded clean read ratios exceeding 91%, with Q20 values above 96.48% and Q30 values above 87.26% (**Supplementary Table 2**). The mapping rates of clean reads to the reference genome and gene set were at least 90.11% and 77.77%, respectively. Specifically, alignment rates to the unique loci of the reference genome and gene set were at least 64.16% and 71.23%, respectively (**Supplementary Table 2**).

**Figure 3:**
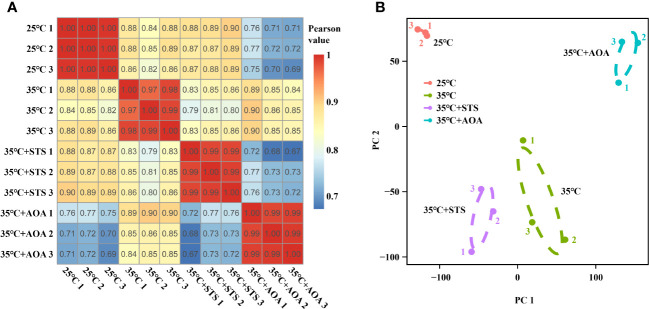
Pearson correlation coefficient and principal component analyses. **(A)** Pearson correlation coefficient analysis for pairwise comparisons of 12 samples across 25°C, 35°C, 35°C+STS, and 35°C+AOA treatments. The Pearson correlation coefficients among biological replicates within each treatment are ≥ 0.97; **(B)** Principal component analysis reveals distinct separation of the four treatments in the plane spanned by PC1 and PC2.

Sequencing saturation analysis revealed that gene identification ratios plateaued when the read number exceeds 50 × 100 K, indicating saturation in the sequencing data for all 12 samples (**Supplementary Figure 3A**). Clustering and correlation analysis, based on gene expression levels, showed tight clustering of biological replicates within each treatment and Pearson correlation coefficients of at least 0.97 between samples within the same treatment (**Figure 3A**; **Supplementary Figure 3B**; **Supplementary Table 3**). PCA demonstrated clear separation of the four treatments along the first two principal components (PC1 and PC2) (**Figure 3B**).

Additionally, transcriptome sequencing encompassed four temperature treatments: 20°C, 25°C, 30°C, and 35°C. Clean read mapping rates to the reference genome and gene set were at least 89.26% and 78.40%, respectively. Notably, alignment rates to unique loci in the reference genome and gene set were at least 87.00% and 71.73%, respectively (**Supplementary Table 2**). Sequencing saturation, clustering, correlation analyses, and PCA were performed for all samples, with all resulting metrics meeting established criteria (**Supplementary Figure 4**).

Overall, the high quality of these samples justifies their use in subsequent analytical procedures.

### 3.4 Screening of differentially expressed genes

**Figure 4:**
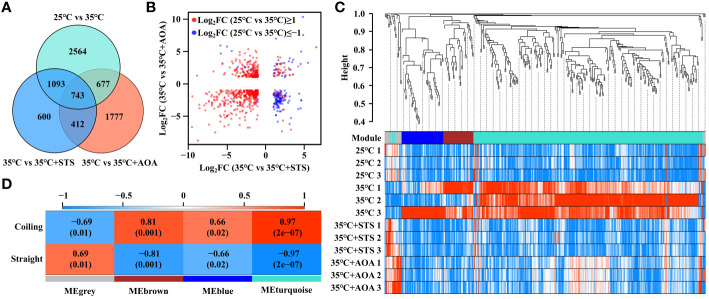
Screening of differentially expressed genes. **(A)** Venn diagram showing the numbers of differentially expressed genes (DEGs) shared among the three comparisons: 25°C vs 35°C, 35°C vs 35°C+STS, and 35°C vs 35°C+AOA. The numbers in the overlapping regions indicate the numbers of DEGs shared by the comparisons; **(B)** Two-dimensional scatter plot of Log_2_FC values for the 743 DEGs in 35°C vs 35°C+STS and 35°C vs 35°C+AOA, with red and blue dots representing DEGs with Log_2_FC(25°C vs 35°C) ≥ 1 and Log_2_FC(25°C vs 35°C) ≤ -1, respectively; **(C)** WGCNA dendrogram showing clustering of the 372 DEGs and the corresponding gene modules. The color bar below the dendrogram indicates the gene modules; **(D)** Heatmap showing the correlation between gene modules and phenotypes. The numbers outside the brackets indicate correlation values, and those inside the brackets indicate *p*-values.

A total of 743 DEGs were shared by 25°C vs 35°C, 35°C vs 35°C+STS, and 35°C vs 35°C+AOA (**Figure 4A**). Among them, 340 genes had Log_2_FC(25°C vs 35°C)≥1, Log_2_FC(35°C vs 35°C+STS)≤-1, and Log_2_FC(35°C vs 35°C+AOA)≤-1; while 32 genes had Log_2_FC(25°C vs 35°C)≤-1, Log_2_FC(35°C vs 35°C+STS)≥1, and Log_2_FC(35°C vs 35°C+AOA)≥1. These 372 genes accounted for approximately 50.07% of the 743 DEGs (**Figure 4B**; **Supplementary Table 4**). Weighted gene co-expression analysis was performed on these 372 DEGs (**Supplementary Table 5**), resulting in three co-expression modules (turquoise, blue, and brown) and a grey module containing unassigned genes. The turquoise module, highly correlated with the root circumnutation phenotype and showing the smallest *p*-value, contained 268 DEGs. Therefore, we focused on the DEGs in this module for further analysis (**Figures 4C, D**; **Supplementary Figure 5**; **Supplementary Table 6**).

### 3.5 GO annotation and classification of DEGs

**Figure 5:**
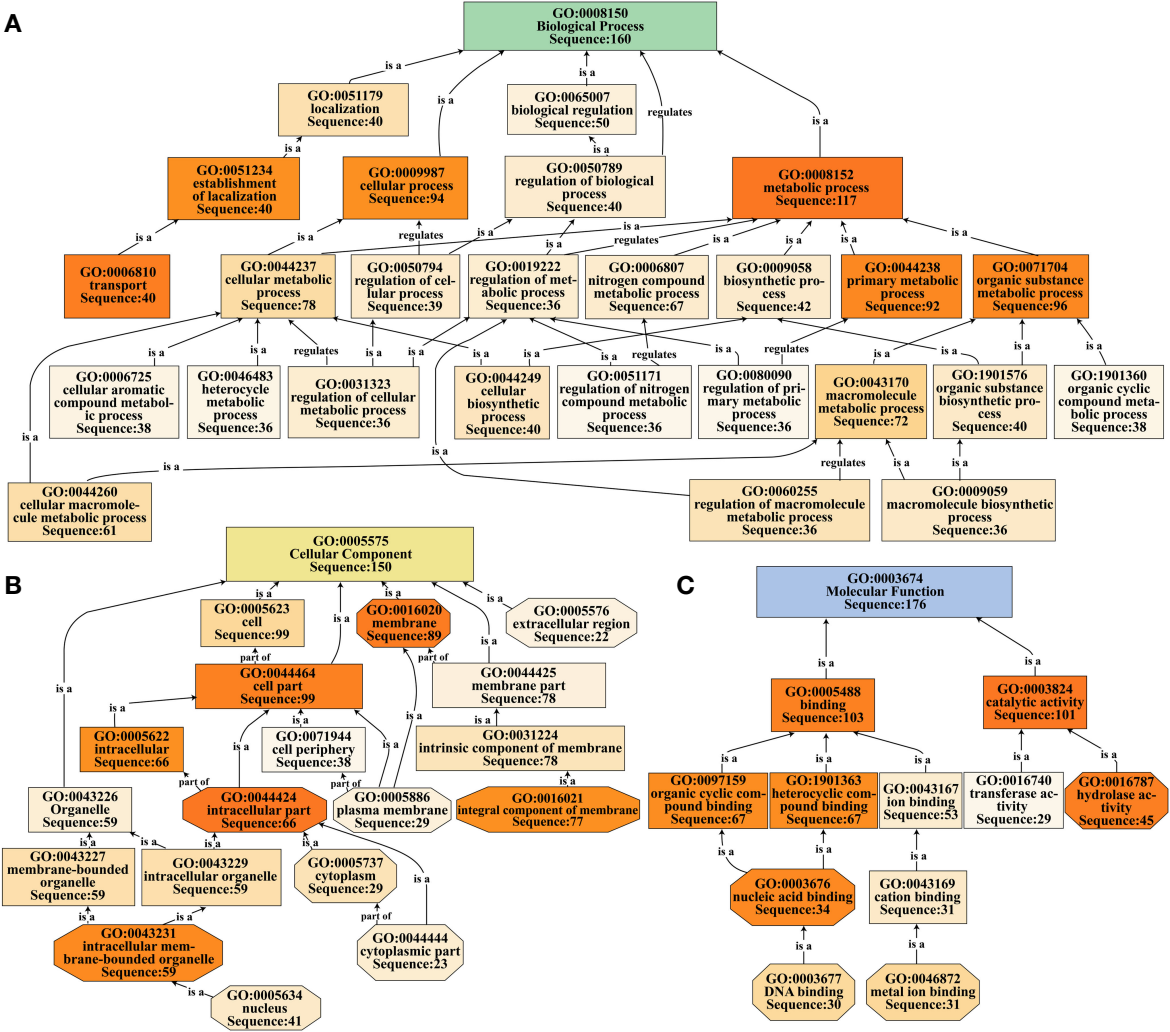
GO functional classification of DEGs in the turquoise module. **(A-C)** Directed acyclic graph representations are used to classify DEGs into three major functional categories: biological process **(A)**, cellular component **(B)**, and molecular function **(C)**. Node colors represent the nodescore values, with darker colors indicating higher nodescore values.

We performed GO functional annotation and classification of 268 DEGs in the turquoise module using the Blast2GO software (**Supplementary Table 7**). The results revealed that the DEGs were predominantly distributed across the three main functional groups: ‘biological process’ (160 DEGs, 59.70%), ‘cellular component’ (150 DEGs, 55.97%), and ‘molecular function’ (176 DEGs, 65.67%). Within the ‘biological process’ category, a notable number of DEGs were associated with ‘metabolic process’ (117 DEGs) and ‘cellular process’ (94 DEGs). Additionally, the ‘transport’ term comprised 40 DEGs, including *OsABCB15* and three *OsNPFs* related to auxin transport (**Figure 5A**; **Supplementary Table 7**, **Supplementary Table 8**). In the ‘cellular component’ category, the DEGs were primarily distributed in the ‘cell part’ (99 DEGs) and ‘membrane’ (89 DEGs) terms, with an additional noteworthy group in the ‘extracellular region’ (22 DEGs) containing genes such as *OsXTH16*, *OsXTH19*, *OsEXPA6*, and *OsEXPA29* involved in cell elongation regulation (**Figure 5B**; **Supplementary Table 7**; **Supplementary Table 8**). Within the ‘molecular function’ category, a significant number of DEGs were identified in the ‘binding’ (103 DEGs) and ‘catalytic activity’ (101 DEGs) terms. Additionally, the ‘hydrolase activity’ and ‘nucleic acid binding’ terms had higher nodescore values, with 45 and 34 DEGs, respectively. Notably, within the ‘catalytic activity’ term, key genes such as *OsYUCCA6* (regulating auxin synthase), *OsEGL1*, *OsXTH16*, and *OsXTH19* (regulating cell elongation), and *OsRMC* (negatively regulating root circumgyration) were identified. Of particular interest was the ‘nucleic acid binding’ term, which included ethylene responsive factor genes such as *OsERF63*, *OsERF81*, *OsERF82*, *OsERF83*, and *OsERF88* (**Figure 5C**; **Supplementary Table 7**, **Supplementary Table 8**).

### 3.6 Expression analysis of genes related to ethylene synthesis and signaling

**Figure 6:**
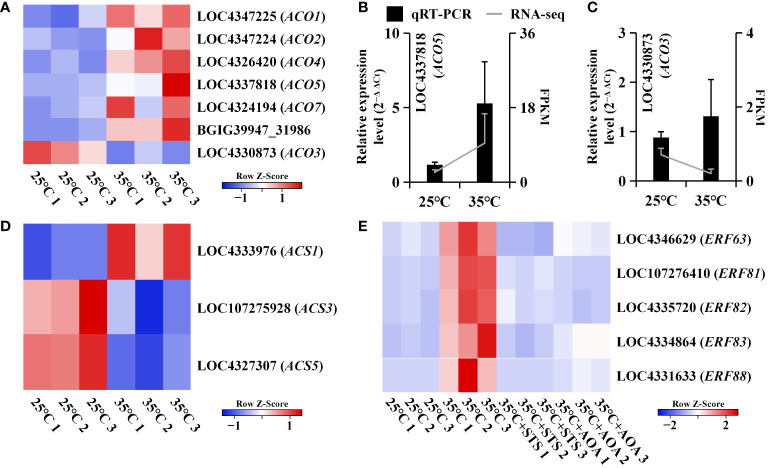
Expression analysis of genes involved in ethylene synthesis and signaling. **(A)** Heatmap shows the expression levels of seven *OsACO* genes at 25°C and 35°C; **(B, C)** qRT-PCR validation of the expression trends of two *OsACO* genes (LOC4337818 and LOC4330873), which are upregulated at 35°C; **(D)** Heatmap shows the expression levels of three *OsACS* genes at 25°C and 35°C; **(E)** Heatmap shows the expression levels of five *OsERF* genes at 25°C, 35°C, 35°C+STS, and 35°C+AOA.

Root circumnutation induced by higher AT was completely eliminated by the application of the ethylene biosynthesis inhibitor AOA or the ethylene perception inhibitor STS, indicating its dependence on the ethylene pathway. Therefore, we focused on the expression changes of genes related to ethylene synthesis and signaling. Ethylene biosynthesis involves two types of rate-limiting enzymes: ACC synthase (ACS) and ACC oxidase (ACO). We identified three *OsACS* genes and seven *OsACO* genes among the DEGs between 25°C and 35°C. Although the expression levels of these three *OsACS* genes were not consistently regulated, the expression levels of the *OsACO* genes, except for *OsACO3*, consistently increased at 35°C and with increasing temperature after 25°C (**Figures 6A-D**; **Supplementary Figure 6A**; **Supplementary Table 9**). Regarding ethylene signaling, we identified five *OsERF* genes in the turquoise gene module, all of which were upregulated at 35°C compared to lower temperatures. However, their expression levels were downregulated at 35°C in the presence of STS or AOA compared to their expression at 35°C without inhibitors. Additionally, their expression increased with increasing temperature after 25°C (**Figure 6E**; **Supplementary Figure 6B**; **Supplementary Table 9**).

### 3.7 Expression analysis of auxin and cell elongation related genes

**Figure 7:**
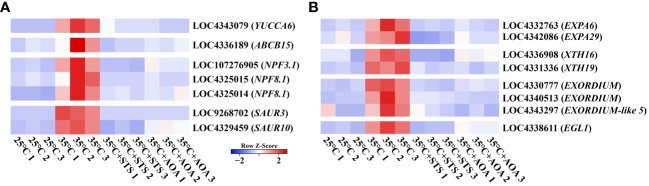
Expression analysis of auxin and cell elongation related genes. **(A)** Heatmap shows the expression levels of *OsYUCCA6*, *OsABCB15*, 3 *OsNPFs*, and 2 *OsSAURs* at 25°C, 35°C, 35°C+STS, and 35°C+AOA; **(B)** Heatmap shows the expression levels of 2 *OsEXPAs*, 2 *OsXTHs*, 3 *OsEXORDIUMs*, and *OsEGL1* at 25°C, 35°C, 35°C+STS, and 35°C+AOA.

Auxin-related and cell elongation-related genes were identified in GO annotations. Auxin regulates plant tropistic growth, and root circumnutation is caused by asymmetric elongation of cells. Therefore, we further investigated the expression changes of specific genes in the turquoise gene module. These included *OsYUCCA6*, responsible for auxin synthesis; *OsABCB15*, *OsNPF3.1*, and two *OsNPF8.1s*, involved in auxin transport; *OsSUAR3* and *OsSUAR10*, mediating auxin response; and *OsEGL1*, two *OsEXPAs*, two *OsXTHs*, and three *OsEXORDIUMs*, associated with cell elongation. Compared to 25°C, the expression of these genes was found to be upregulated at 35°C. However, their expression was reduced at 35°C in the presence of STS or AOA compared to the high expression levels observed at 35°C without inhibitors. Additionally, these genes exhibited an upregulation pattern with increasing temperatures above 20°C (**Supplementary Figures 7A, B**; **Supplementary Table 10**). Therefore, the expression of these genes is upregulated by increasing AT, and this upregulation is mediated through the ethylene pathway (**Figures 7A, B**; **Supplementary Table 10**).

### 3.8 Weighted gene co-expression network

**Figure 8:**
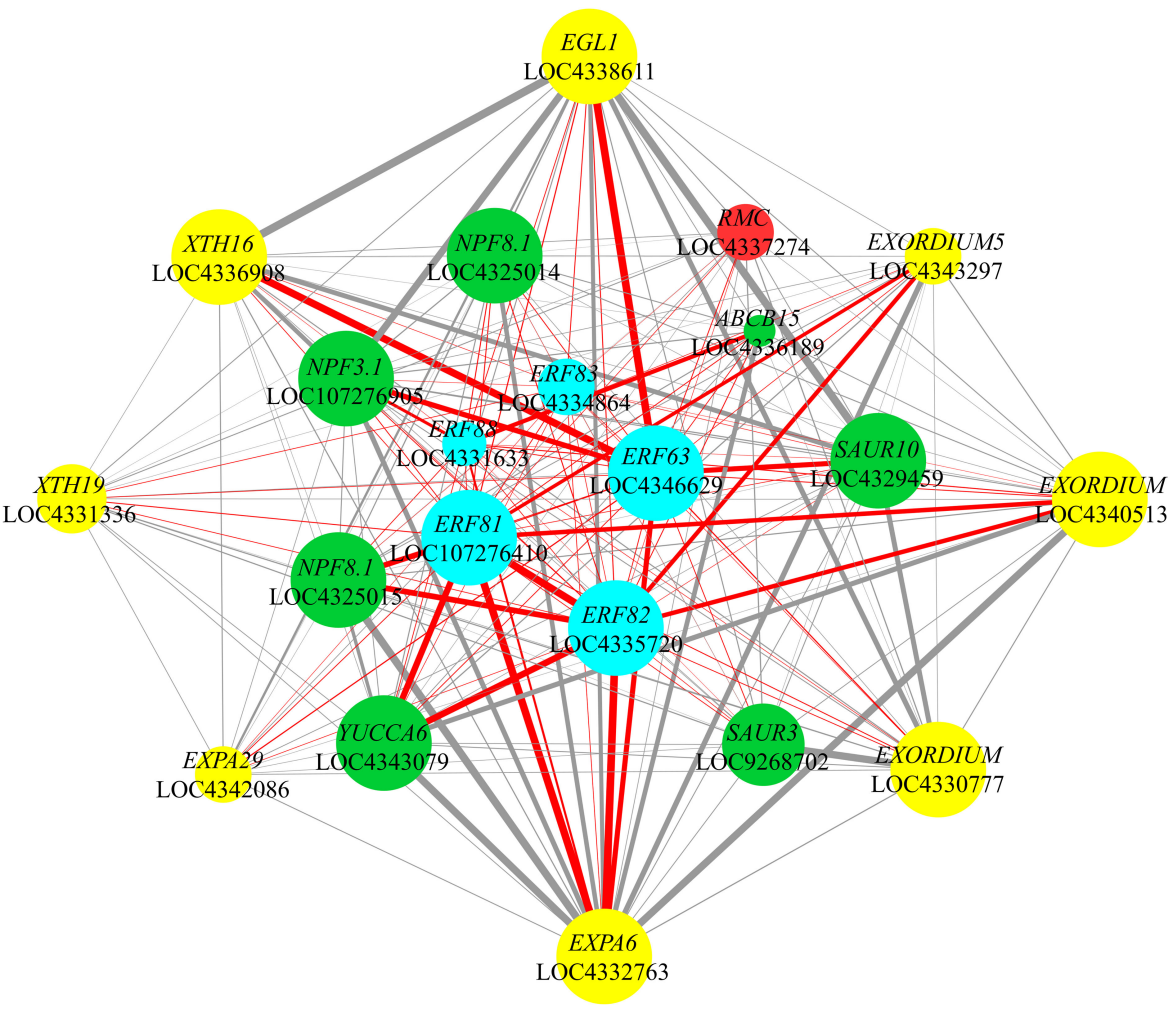
Co-expression network of 21 key genes. The co-expression relationship among *OsERFs*, genes related to auxin and cell elongation, and *OsRMC* is presented. The sizes of the nodes represent the degree values of the genes, with larger nodes indicating higher degrees. The thickness and darkness of the edges correspond to the weight values between genes, where thicker and darker edges denote stronger weights. Cyan, green, yellow, and red nodes represent *OsERFs*, auxin-related genes, cell elongation-related genes, and *OsRMC*, respectively. Additionally, edges connected specifically to *OsERFs* are highlighted in red.

Based on previous analyses, we focused on the co-expression relationships among five ethylene response factor genes (*OsERF63*, *OsERF81*, *OsERF82*, *OsERF83*, and *OsERF88*), the auxin synthesis enzyme gene *OsYUCCA6*, auxin transport genes *OsABCB15* and *OsNPFs*, auxin-responsive genes *OsSAURs*, cell elongation-related genes (*OsEGL1*, *OsXTHs*, *OsEXPAs*, and *OsEXORDIUMs*), alongside *OsRMC*, a negative regulator of root circumnutation (**Figure 8**).

Within the turquoise co-expression module, a remarkable connectivity was observed among *OsERF63*, *OsERF81*, and *OsERF82*, with these genes being linked to 95.88%, 96.63%, and 93.26% of the nodes, respectively. Notably, *OsERF63* exhibited an extensive co-expression profile, encompassing all the focal genes. Considering co-expression relationships with weight values exceeding 0.3, we noted the co-expression of *OsERF81* and *OsERF82* with *OsYUCCA6*, as well as that of *OsERF82* with *OsNPF8.1* (LOC4325015). This underscores a substantial interplay between ethylene-responsive factor genes and both the auxin synthesis enzyme gene and auxin transport genes. With weight values surpassing 0.3, *OsERF63* demonstrated co-expression with *OsEGL1* and *OsXTH16*, while *OsERF81* and *OsERF82* exhibited co-expression with *OsEXPA6*. These findings suggest a robust correlation between ethylene response factor genes and cell elongation-related genes. Furthermore, the weight value exceeding 0.25 for the co-expression relationship between *OsYUCCA6* and both *OsEXPA6* and *OsEXORDIUM* (LOC4340513) highlights a significant interplay between the auxin synthesis enzyme gene and genes governing cell elongation. Among the pronounced co-expression relationships observed between auxin transport genes and cell elongation-related genes, three *OsNPFs* and *OsABCB15* were found to be co-expressed with *OsEXPA6*. Additionally, co-expression relationships were observed between *OsNPF3.1* and both *OsXTH16* and *OsEGL1*, with all weight values surpassing 0.25. Notably, the co-expression relationships between *OsSAUR3* and *OsEXORDIUM* (LOC4330777), as well as between *OsSAUR10* and *OsEGL1*, exhibited weight values exceeding 0.3, emphasizing a marked correlation between auxin-responsive genes and cell elongation-related genes (**Figure 8**; **Supplementary Figure 8**; **Supplementary Table 11**).

Moreover, *OsRMC*, a gene negatively regulating root circumnutation, exhibited distinct co-expression relationships with *OsERF63*, *OsERF81*, *OsERF82*, and *OsERF83*. Among these relationships, the co-expression between *OsRMC* and *OsERF63* stood out with the highest weight value of 0.16 (**Figure 8**; **Supplementary Figure 8**; **Supplementary Table 11**). This underscores a notable interplay between the gene negatively modulating root circumnutation and the ethylene response factor genes.

### 3.9 Validation of transcriptome sequencing data by qRT-PCR

**Figure 9:**
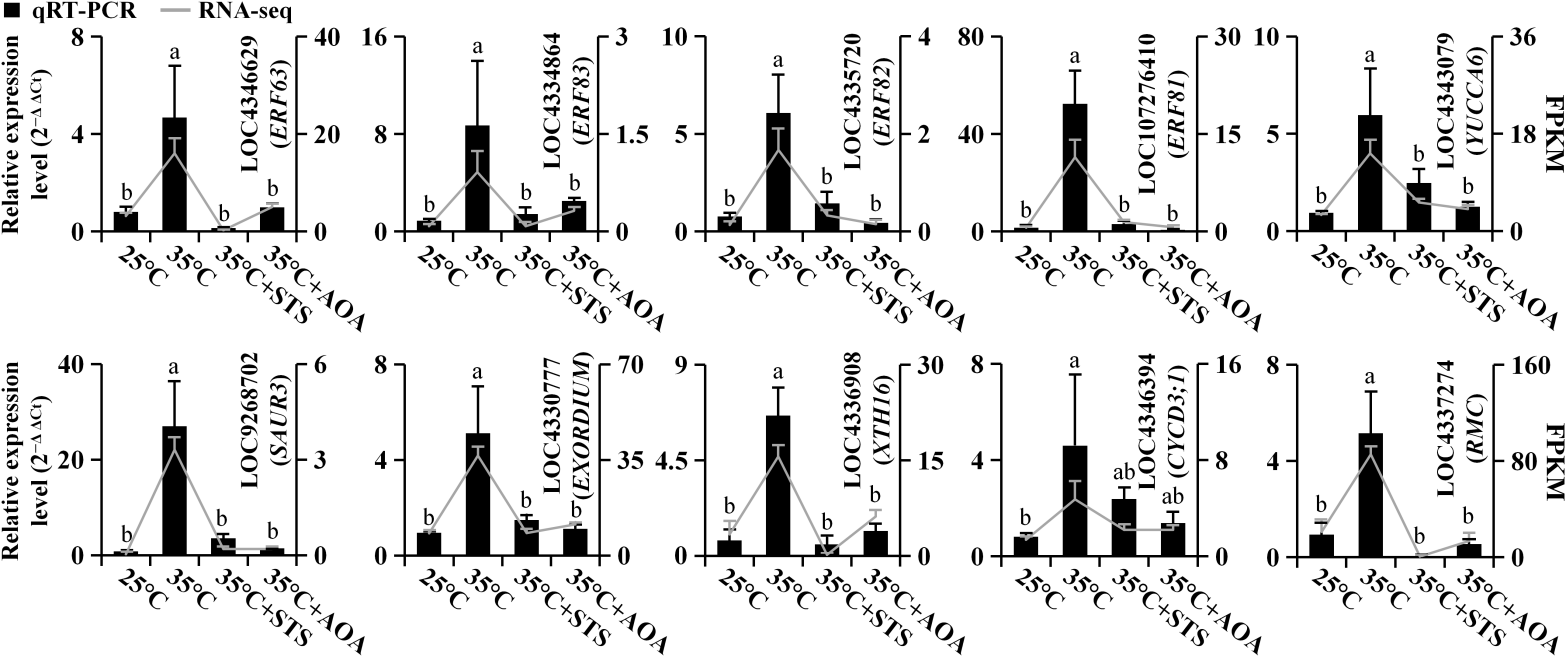
Real-time quantitative polymerase chain reaction. Ten genes were selected for qRT-PCR to validate the transcriptome sequencing data. The qRT-PCR results are represented by bar charts, while the transcriptome sequencing results are represented by line charts. Different lowercase letters indicate significant differences in qRT-PCR results among the corresponding treatments (*p* < 0.05).

We selected 10 genes from the turquoise module for validation by qRT-PCR (in the 25°C, 35°C, 35°C+STS, and 35°C+AOA treatments). These genes comprised 4 ethylene response factor genes (*OsERF63*, *OsERF81*, *OsERF82*, and *OsERF83*), 2 auxin-related genes (*OsYUCCA6* and *OsSAUR3*), 2 cell elongation-related genes (*OsEXORDIUM* (LOC4330777) and *OsXTH16*), a cell division-related gene (*OsCYCD3;1* (LOC4346394)), and *OsRMC*, which inhibits root curling. The qRT-PCR results for all 10 genes correlated with the transcriptome sequencing data, indicating the reliability of the latter (**Figure 9**; **Supplementary Table 12**). Additionally, nine genes were selected from the 12 samples under varying temperature treatments (20°C, 25°C, 30°C, and 35°C), including ACC oxidase genes (*OsACO5* and *OsACO7*). qRT-PCR validation was performed for all nine genes, and the results were consistent with the transcriptome sequencing data, confirming the reliability of the transcriptome sequencing data (**Supplementary Figure 9**; **Supplementary Table 12**).

## 4 Discussion

Different ranges of AT had distinct effects on root circumnutation and elongation growth. In this study, when the temperature was raised from 20°C to 25°C, the roots did not exhibit circumnutation, but their elongation increased significantly. In contrast, when the temperature was increased from 30°C to 35°C, root circumnutation increased significantly, but there was no significant change in root elongation. Previous studies have reported that *Arabidopsis* plants with the *npf7.3* mutation or cultivated on a horizontal plate with nitrate deficiency, as well as rice plants cultured with exogenous application of BL, exhibited a root circumnutation phenotype, while root length did not show significant changes (Buer et al., 2003; Jiang et al., 2007; Chen et al., 2015; Cai et al., 2021b). Therefore, root circumnutation and elongation growth appear to be two relatively independent processes.

Sufficient evidence has been reported showing that higher AT promotes ethylene biosynthesis in plants. For instance, *Arabidopsis* seedlings grown at 27°C produce more ethylene than those grown at 22°C (Fei et al., 2017); high-temperature treatment increases ethylene production rates in leaves and pods of soybean (*Glycine max*) (Maduraimuthu and Prasad, 2010); ethylene synthesis is increased in wheat (*Triticum aestivum*) (40°C) and tomato (*Solanum lycopersicum*) (42°C) seedlings under high-temperature stress (Khan et al., 2013; Pan et al., 2019; Poór et al., 2022); and ethylene levels in rice leaves are significantly increased at 40°C (Gautam et al., 2022). The increase in ethylene biosynthesis can be attributed to two factors: an increase in the expression of ethylene biosynthesis enzyme genes and an increase in the activity of ethylene biosynthesis enzymes. Regarding gene expression, although the expression of *OsACSs* in this study was irregular, the expression of *OsACOs* consistently showed upregulation at 35°C (**Supplementary Figure 6**; **Supplementary Table 9**). As for enzyme activity, a study reported that wheat increases ethylene biosynthesis by increasing ACS activity under high-temperature stress (40°C) (Khan et al., 2013). This suggests that higher AT may also promote ethylene biosynthesis by enhancing ACS activity, thereby promoting root circumnutation.

In terms of ethylene signal transduction, *OsERF63*, *OsERF81*, and *OsERF82* have been identified as key genes in ethylene-induced root circumnutation (Cai et al., 2021b). In this study, all three *OsERFs* were identified as core transcription factor genes involved in AT-induced root circumnutation through the ethylene pathway. Among them, *OsERF82* has also been identified as a key player in BR-induced rice root circumnutation through the ethylene pathway, and its expression is upregulated by BR (Cai et al., 2021a). However, current research on the functionality of these three *OsERFs* is limited to their response to stress, with no reports exploring their specific roles. *OsERF63* is upregulated under heat stress and hypoxic conditions, while *OsERF82* is upregulated under salt stress (Kumar et al., 2020; Liu et al., 2020; Wang et al., 2021). The involvement of these three *OsERFs* in root circumnutation may not be coincidental, and their role in this process deserves further exploration.

Auxin biosynthesis and transport are important factors regulating plant directional growth. Regarding auxin biosynthesis, we identified *OsYUCCA6* as the rate-limiting enzyme gene in the tryptophan-dependent auxin biosynthesis pathway (Cao et al., 2019). Concerning auxin transport, we identified *OsABCB15*, which is also a core gene involved in ethylene-induced root circumnutation that we previously investigated (Cai et al., 2021b). We also identified three *OsNPFs*, which encode proteins belonging to the NITRATE TRANSPORTER 1/PEPTIDE TRANSPORTER family. Although some members of this family were originally characterized for their roles in nitrate or di/tripeptide transport, recent studies have shown that specific members, such as *AtNPF5.12*, *AtNPF6.3*, and *AtNPF7.3*, are also implicated in auxin transport (Krouk et al., 2010; Michniewicz et al., 2019; Watanabe et al., 2020). Additionally, it has been demonstrated that elevated temperatures can induce the accumulation of AtPIN2 on the plasma membrane, thereby enhancing auxin polar transport in roots (Hanzawa et al., 2013). This suggests that higher AT may regulate root circumnutation by altering the subcellular localization of auxin transporters.

In terms of genes related to cell elongation, we particularly focused on *OsEXPA6*, *OsEXPA29*, *OsXTH16*, *OsXTH19*, *OsEGL1*, and *OsEXORDIUMs*. As members of the α-EXPANSIN family, EXPAs can mediate pH-dependent cell wall extension by disrupting the bonding of the glycans to the microfibril surface or to each other, thereby promoting cell elongation (Cosgrove, 2000; Choi et al., 2006). *OsXTHs* encode xyloglucan endotransglucosylase and/or hydrolases, which can split and reconnect xyloglucan crosslinks in the cell wall and play an important role in regulating cell expansion (Yokoyama et al., 2004; Nishitani, 2005; Hara et al., 2014). *OsEGL1* encodes endo-(1,3;1,4)-β-glucanase, which may promote cell wall loosening by hydrolyzing (1,3;1,4)-β-glucan in the cell wall matrix (Akiyama et al., 2009). EXORDIUM is located in the cell wall and mediates cell expansion downstream of the BR signal (Schröder et al., 2009). In this study, the expression levels of these genes were upregulated at 35°C and subsequently downregulated by an ethylene inhibitor, indicating that higher AT may regulate root cell elongation through the ethylene pathway. Subsequent studies could further investigate the differential expression levels of these genes in inner and outer cortical cells of the root tip.

*OsRMC* was initially identified as a negative regulator in JA-induced rice root circumnutation and was named accordingly based on its function (Jiang et al., 2007; Lourenço et al., 2015). It consists of a signal peptide and two Cysteine-rich repeat domains, lacking transmembrane regions and kinase domains, and belongs to the Cysteine-rich repeat secretory proteins (Chen, 2001). Studies have shown that the expression of *OsRMC* is directly regulated by OsEREBP1 and OsEREBP2 (AP2/ERF transcription factors) (Serra et al., 2013; Lourenço et al., 2015). In ethylene-induced root circumnutation, *OsRMC* is upregulated (Cai et al., 2021b). In this study, *OsRMC* was also upregulated in AT-induced rice root circumnutation, which depended on the ethylene pathway. This negative regulator is involved in both ethylene and AT-induced root circumnutation. Recent research has shown that OsRMC slightly inhibits the activity of ascorbate peroxidases (APXs) and promotes the accumulation of reactive oxygen species (ROS) (Wang et al., 2022). Other studies have shown that ROS can enhance plant root gravitropism, and exogenous application of H_2_O_2_ can inhibit root circumnutation to a certain extent (Krieger et al., 2016; Orman-Ligeza et al., 2016). This raises the question of whether the negative regulation of OsRMC on root circumnutation is mediated by promoting the accumulation of ROS at the root tip, which warrants further investigation.

In summary, this study demonstrates for the first time that higher AT can induce rice root circumnutation by promoting ethylene biosynthesis and signaling. In this process, AT promotes the expression of a series of genes through the ethylene pathway, including those involved in auxin synthesis and transport (such as *OsYUCCA6*, *OsABCB15*, *OsNPFs*) and those regulating cell elongation (such as *OsEXPAs*, *OsXTHs*, *OsEGL1*, *OsEXORDIUMs*), ultimately leading to root circumnutation. Meanwhile, *OsRMC* plays a negative regulatory role in this process (**Supplementary Figure 10**). The identification of these core genes provides a basis for uncovering the molecular mechanism by which AT induces root circumnutation through the ethylene pathway.

## Data availability statement

The datasets presented in this study can be found in online repositories. The names of the repository/repositories and accession number(s) can be found in the article/**Supplementary Material**.

## Author contributions

ZC: Writing – original draft, Writing – review & editing, Conceptualization, Investigation, Data curation, Methodology, Supervision, Formal analysis, Project administration, Validation, Funding acquisition, Resources, Visualization. YD: Writing – original draft, Writing – review & editing, Conceptualization, Investigation, Data curation, Formal analysis, Validation, Visualization. XJ: Writing – original draft, Conceptualization, Investigation, Data curation, Methodology, Supervision, Formal analysis. HX: Writing – original draft, Investigation, Data curation. ZH: Writing – original draft, Conceptualization, Investigation, Data curation, Methodology. ZX: Writing – original draft, Conceptualization, Investigation, Data curation. XY: Writing – review & editing, Funding acquisition. JL: Conceptualization, Methodology, Project administration, Writing – review & editing, Funding acquisition, Resources.
