# Supplementary material for: Ambient temperature regulates root circumnutation in rice through the ethylene pathway: transcriptome analysis reveals key genes involved
Source: Front Plant Sci. 2024 Mar 8;15:1348295. doi: 10.3389/fpls.2024.1348295 (PMC10957643; doi:10.3389/fpls.2024.1348295)
Supplement: Supplementary file 2 [file DataSheet_4.pdf]

Legends to Supplementary Figures 1-10

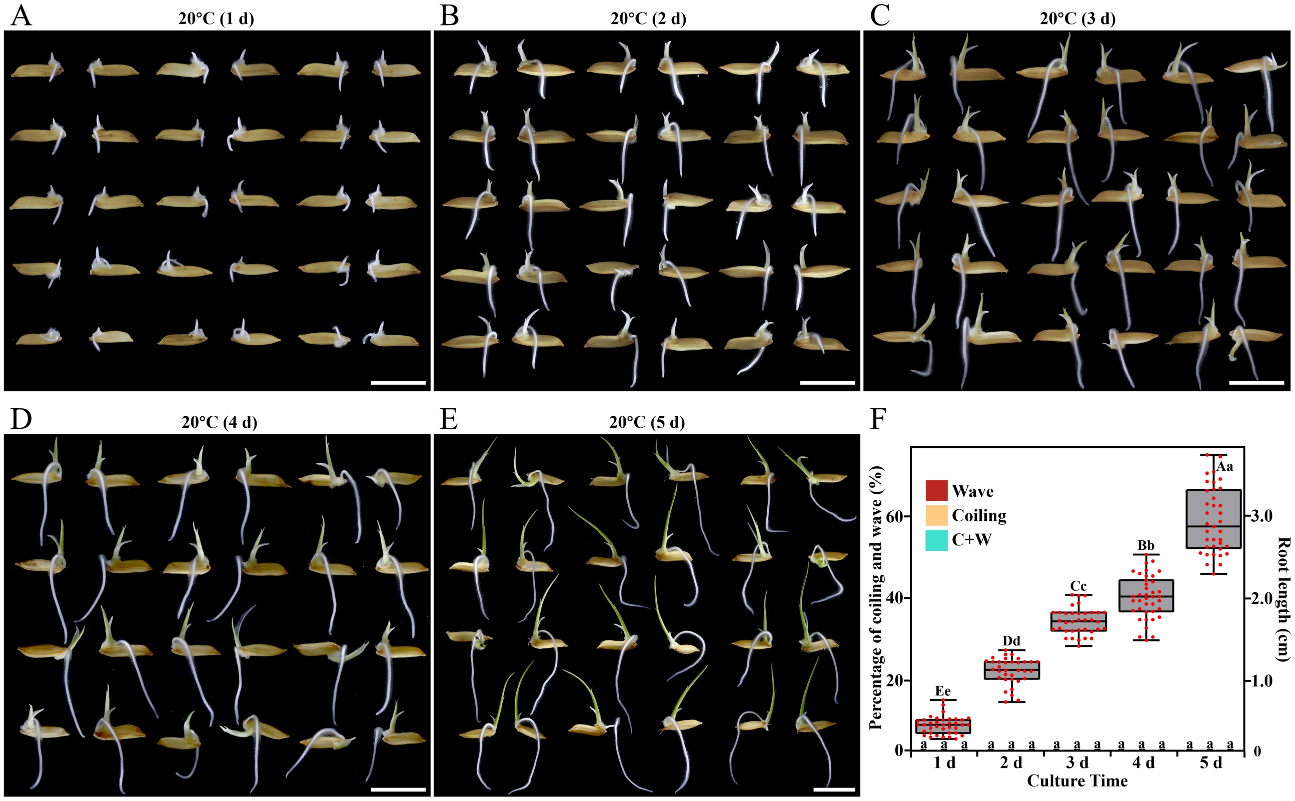

Supplementary Figure 1. Rice root growth at 20°C.

(**A**)-(**E**): The growth status of rice roots after cultivation at 20°C for 1-5 days, respectively; (**F**): Root circumnutation percentages and root lengths from day 1 to day 5; scale bar = 1 cm.

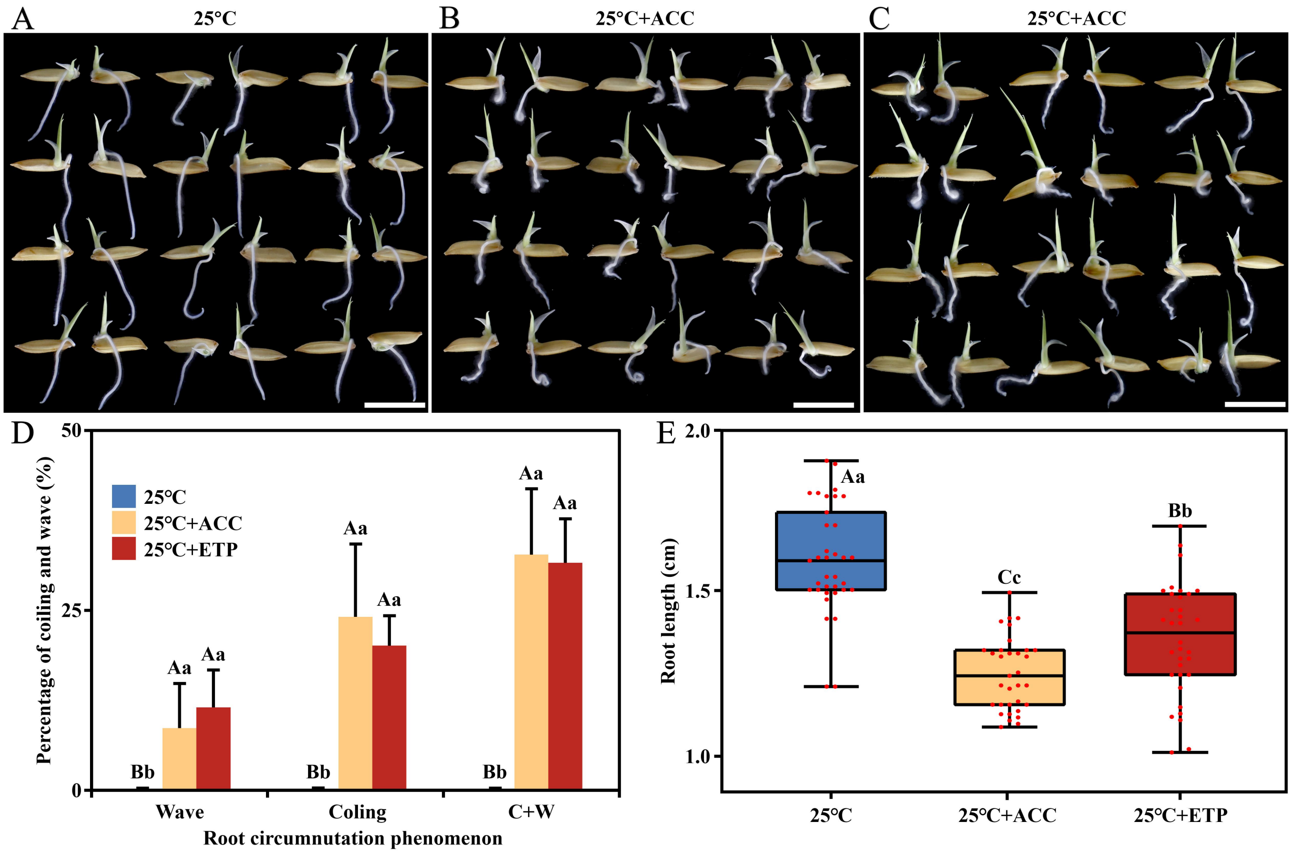

Supplementary Figure 2. Ethylene-induced rice root circumnutation.

(**A**)-(**C**): Rice root growth under three treatments: 25°C (A), 25°C + ACC (B), and 25°C + ETP (C); (D)-(E): Root circumnutation percentages (D) and root lengths (E) under the aforementioned three treatments; scale bar = 1 cm, and the cultivation time was 2 days.

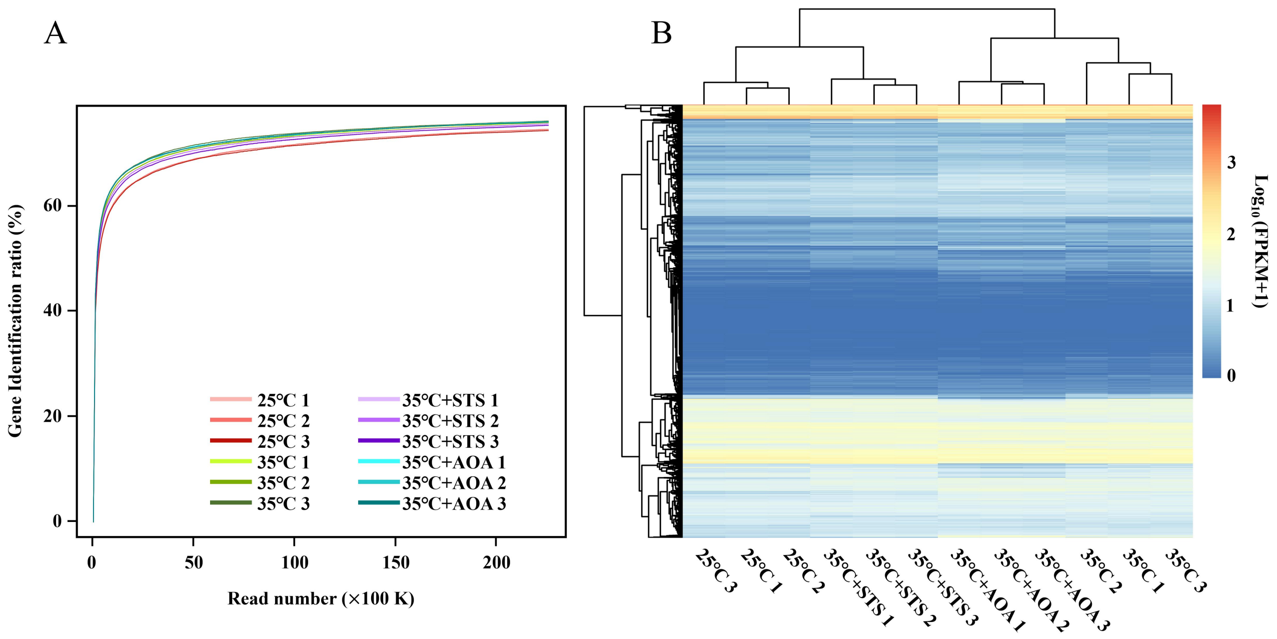

Supplementary Figure 3. Sequencing saturation analysis and sample clustering of the 12 samples.

(**A**): Sequencing saturation analysis shows that as the "Read number" increases, the gene identification ratios of the 12 samples also increase. When the "Read number" exceeds 50 × 100 K, the increase in the gene identification ratio for each sample flattens, indicating that the sequencing data for all 12 samples have reached saturation. (**B**): Sample clustering of 12 samples based on gene expression levels under 25°C, 35°C, 35°C+STS, and 35°C+AOA treatments. The three biological replicates of each treatment are clustered together.

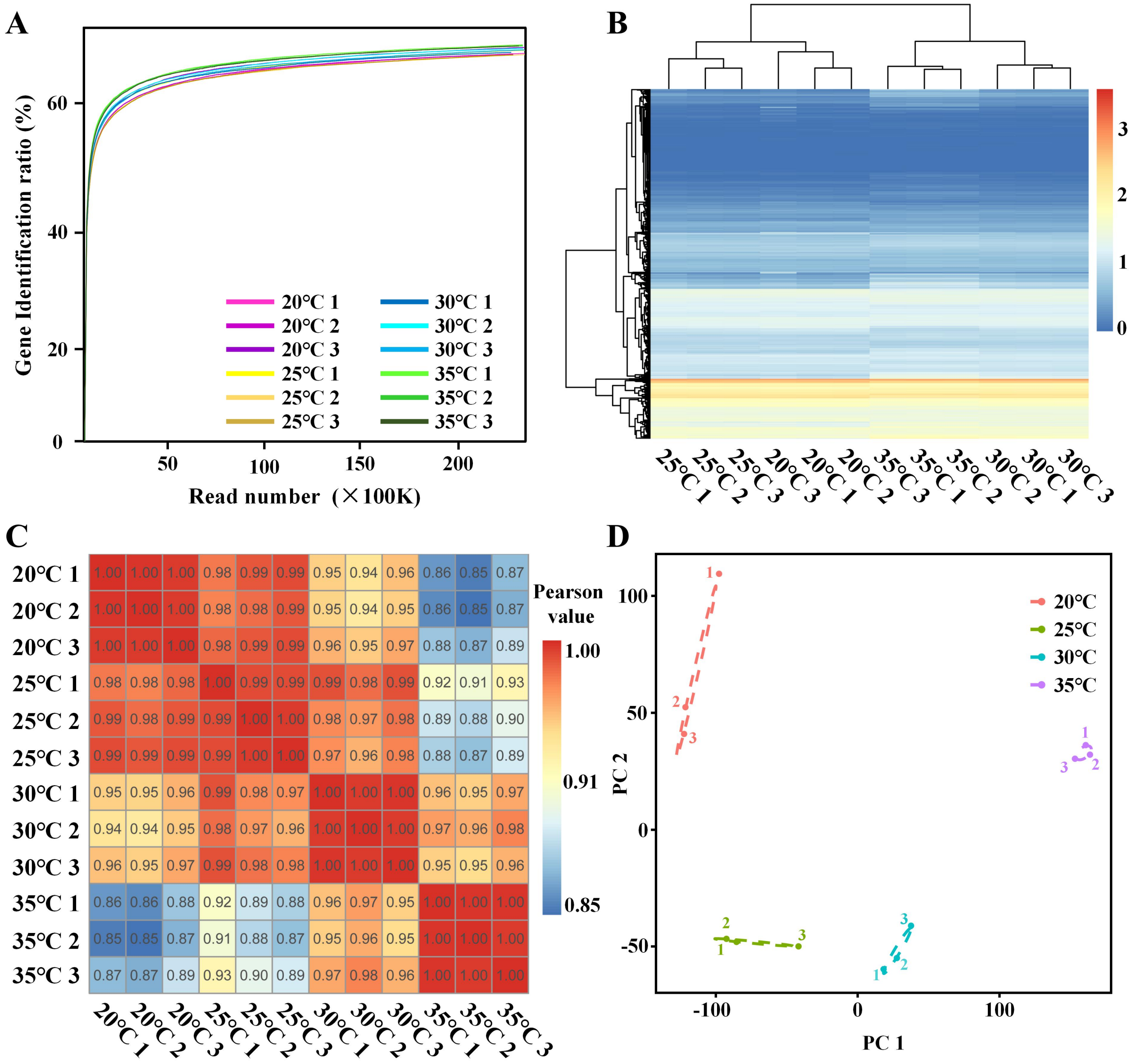

Supplementary Figure 4. Sequencing saturation, sample clustering, Pearson correlation coefficient, and principal component analyses.

(**A**): Sequencing saturation analysis shows that as the "Read number" increases, the gene identification ratios also increase. When the "Read number" exceeds 50 × 100 K, the increase in the gene identification ratio for each sample flattens, indicating that the sequencing data for all 12 samples have reached saturation. (**B**): Sample clustering of 12 samples based on gene expression levels under 20°C, 25°C, 30°C, and 35°C treatments. The three biological replicates of each treatment are clustered together. (**C**): Pearson correlation coefficient analysis for pairwise comparisons of 12 samples under 20°C, 25°C, 30°C, and 35°C treatments. Pearson correlation coefficients among biological replicates are ≥ 0.99. (**D**): Principal component analysis shows that the four treatments are separated in the plane formed by PC1 and PC2.

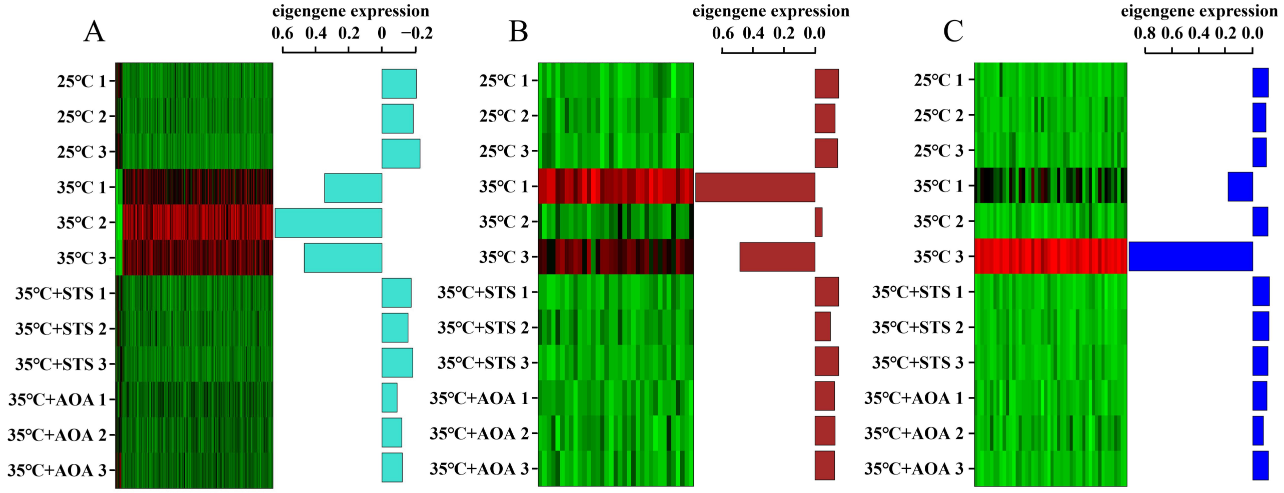

Supplementary Figure 5. Expression profiles of module genes in 25°C, 35°C, 35°C+STS, and 35°C+AOA treatments.

(**A**)-(**C**): Heatmaps show the expression levels of genes in the turquoise (**A**), brown (**B**), and blue (**C**) modules across 12 samples (left) and the expression levels of their corresponding eigengenes (right).

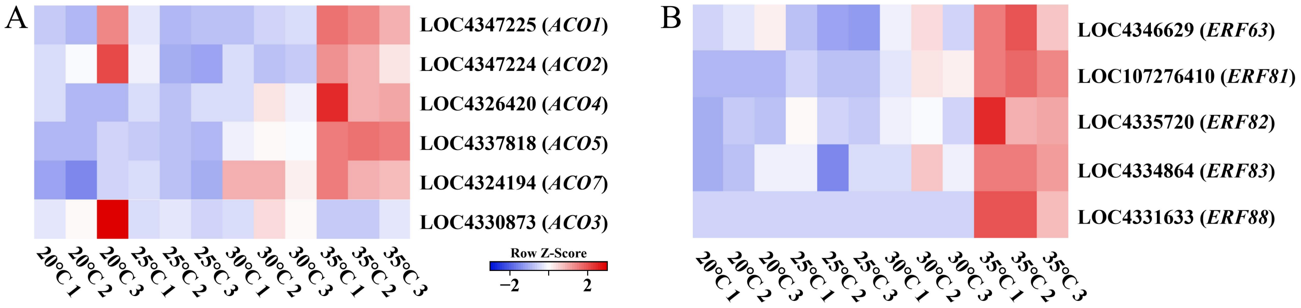

Supplementary Figure 6. Expression changes of genes related to ethylene synthesis and signaling with elevated ambient temperature levels.

(**A**): Heatmap showing the expression levels of six *OsACO* genes at 20°C, 25°C, 30°C, and 35°C. (**B**): Heatmap showing the expression levels of five *OsERF* genes at 20°C, 25°C, 30°C, and 35°C.

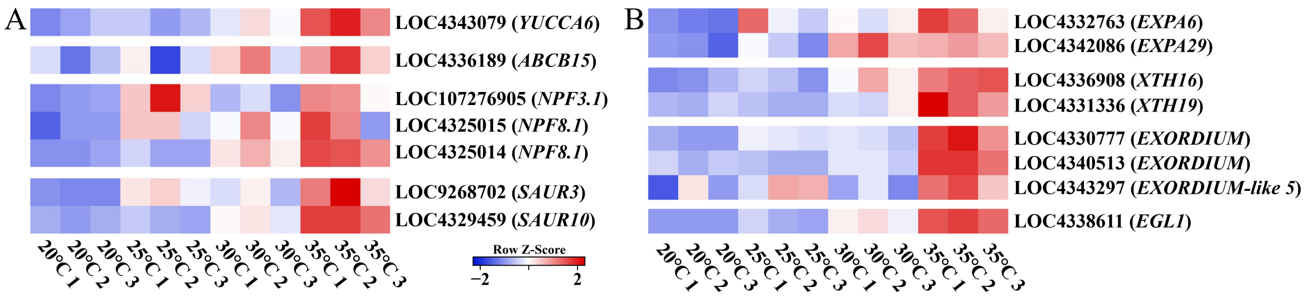

Supplementary Figure 7. Expression changes of genes related to auxin and cell elongation with elevated ambient temperature levels.

(A): Heatmap showing the expression levels of *OsYUCCA6*, *OsABCB15*, 3 *OsNPFs*, and 2 *OsSAURs* at 20°C, 25°C, 30°C, and 35°C. (B): Heatmap showing the expression levels of 2 *OsEXPAs*, 2 *OsXTHs*, 3 *OsEXORDIUMs*, and *OsEGL1* at 20°C, 25°C, 30°C, and 35°C.

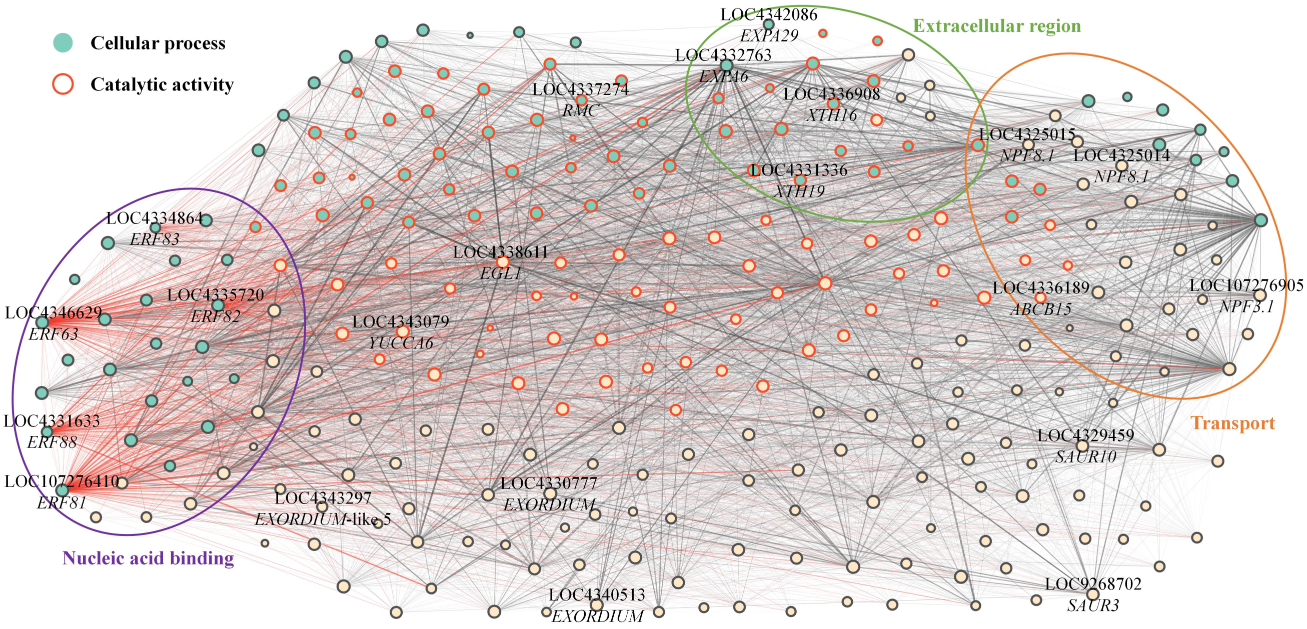

Supplementary Figure 8. Co-expression network of turquoise module genes.

The co-expression relationship among 268 turquoise module genes is illustrated. The sizes of the nodes indicate the degree values of the genes, with larger nodes representing higher degrees. The thickness and darkness of the edges correspond to the weight values between genes, with thicker and darker edges indicating greater weights. The green nodes represent genes associated with the "cellular process" term, while nodes with red outer circles represent genes related to the "catalytic activity" term. The genes located within the purple, green, and orange oval regions belong to the "nucleic acid binding", "extracellular region", and "transport" terms, respectively. Additionally, the edges connected to *OsERFs* are highlighted in red.

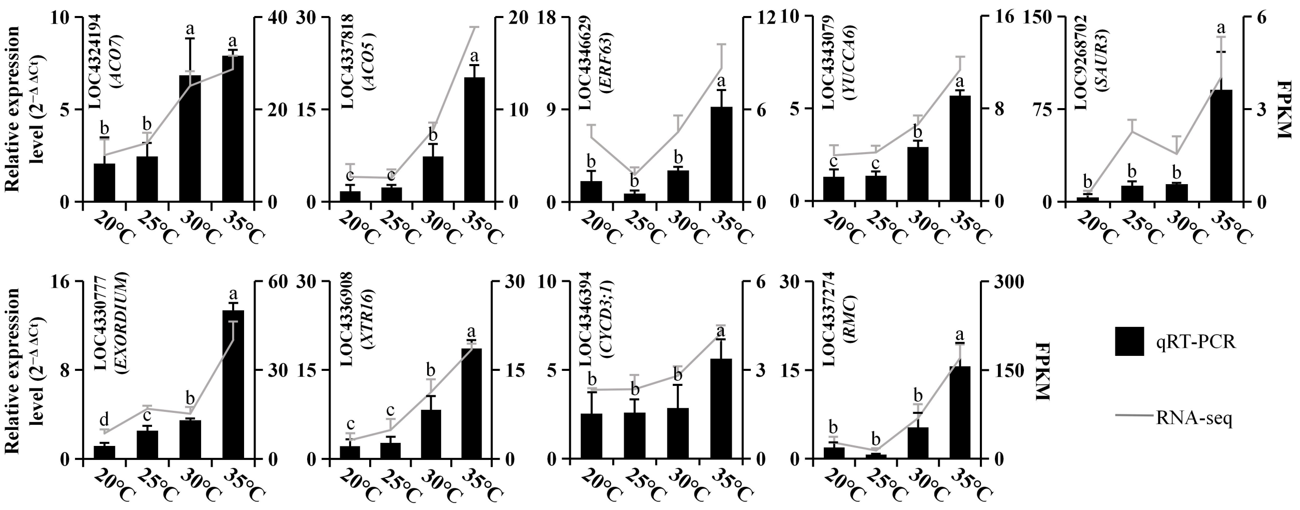

Supplementary Figure 9. qRT-PCR validation of gene expression changes in response to increasing ambient temperatures.

Nine genes were selected for qRT-PCR analysis to validate the transcriptome sequencing data. The qRT-PCR results and transcriptome sequencing data are represented by bar charts and line charts, respectively. Different lowercase letters indicate significant differences in the qRT-PCR results among the corresponding treatments ($p<0.05$).

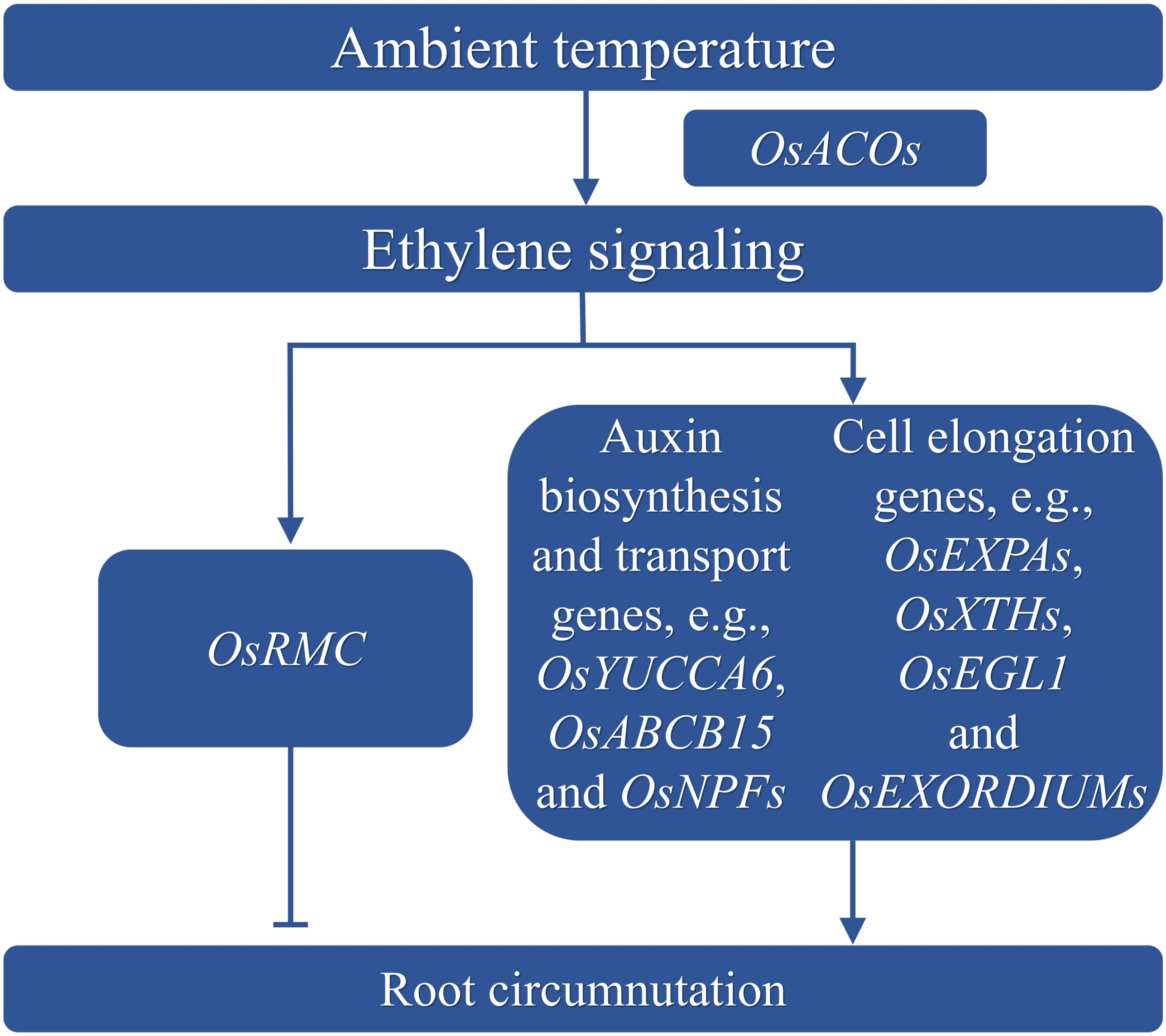

Supplementary Figure 10. Molecular model of root circumnutation induced by higher ambient temperature through the ethylene pathway.

Higher AT upregulates the expression of a series of genes by promoting the biosynthesis and signaling of ethylene. These include genes involved in the synthesis and transport of auxin (such as *OsYUCCA6*, *OsABCB15*, and *OsNPFs*), as well as genes involved in cell elongation (such as *OsEXPAs*, *OsXTHs*, *OsEGL1*, and *OsEXORDIUMs*), ultimately leading to root circumnutation. Meanwhile, *OsRMC* plays a negative regulatory role in this process.
