# Supplementary material for: Ambient temperature regulates root circumnutation in rice through the ethylene pathway: transcriptome analysis reveals key genes involved
Source: Front Plant Sci. 2024 Mar 8;15:1348295. doi: 10.3389/fpls.2024.1348295 (PMC10957643; doi:10.3389/fpls.2024.1348295)
Supplement: Supplementary file 3 [file DataSheet_5.pdf]

frontiers

Supplementary Tables 1-12 guidance

Supplementary Table 1 Information on genes selected for qRT-PCR analysis

Supplementary Table 2 Summary of transcriptome data

Supplementary Table 3 Expression profiles of all individual genes in 12 samples under treatments of 25°C, 35°C, 35°C+STS, and 35°C+AOA

Supplementary Table 4 743 DEGs in the comparison groups of 25°C vs 35°C, 35°C vs 35°C+STS, and 35°C vs 35°C+AOA

Supplementary Table 5 Expression profiles of 372 DEGs for WGCNA

Supplementary Table 6 Genes in 3 modules output by WGCNA

Supplementary Table 7 GO function classification of 268 DEGs in turquoise module

Supplementary Table 8 The corresponding gene names, sources, and sequences in turquoise module

Supplementary Table 9 Expression profiles of the ethylene synthase genes and ethylene response factor genes

Supplementary Table 10. Expression profiles of genes related to auxin and genes regulating cell elongation

Supplementary Table 11. Co-expression network of 268 genes in turquoise module

Supplementary Table 12. Processing and analysis of qRT-PCR data
